# Interaction of cCMP with the cGK, cAK and MAPK kinases in murine tissues

**DOI:** 10.1186/2050-6511-16-S1-A101

**Published:** 2015-09-02

**Authors:** Stefanie Wolfertstetter, Frank Schwede, Franz Hofmann, Jens Schlossmann

**Affiliations:** 1 Department of Pharmacology and Toxicology, University of Regensburg, Germany; 2 Biolog Life Science Institute, Bremen, Germany; 3 Carvas, Institute for Pharmacology and Toxicology, TU Munich, Munich, Germany

## Background

cAMP and cGMP are well established second messengers that are essential for numerous of (patho)physiological processes. These purine cyclic nucleotides activate cAK and cGK, respectively. So far, there was no evidence of further cyclic nucleotides acting as second messengers. Meanwhile the existence of cCMP was described [1,2]. cCMP activates the purified cyclic nucleotide-dependent protein kinases cAK and cGK and induces relaxation of vascular smooth muscle via cGKI [3]. Furthermore, it was postulated that cCMP is relevant for cell growth [4] and blood cell function [5]. However, functions regulated by cCMP are mostly unknown.

## Methods and results

To elucidate propable functions cCMP-binding and -activated proteins were identified using different methods. Competitive binding assays identified cAK, cGKI, and cGKII as cCMP-binding proteins in murine tissue lysates, using 4-AH-cCMP agarose. cCMP (200 µM) was added (+) or omitted (-) during the affinity chromatography experiments to investigate the specificity of the binding. An interaction between cCMP/MAPK and a protein-protein complex of MAPK/cGK were detected via cCMP affinity chromatography and co-immunoprecipitation, respectively. Interestingly, no specific interaction of MAPK with 8-AET-cGMP agarose was detected. Moreover, DB-cCMP (100 µM) was also able to stimulate the phosphorylation of p44/p42 MAPK. The phosphorylation of MAPK was inhibited by the addition of the PKA inhibitor AS_5-24_, suggesting a stimulatory function for PKA in cCMP-mediated MAPK phosphorylation. To elucidate the role of cGK in murine tissues in this process, we used cGKII knockout (cGKII KO) and cGKI knockout (cGKI KO) mice. We detected stimulation in the jejunum tissues from cGKI KO and cGKII KO mice. It is interesting to note that the phosphorylation in the jejunum cGKII KO tissue was significantly increased when compared with the WT and cGKI KO tissues, suggesting an inhibitory role for cGKII in cCMP-induced MAPK phosphorylation in the jejunum.

## Conclusion

These results suggest that MAPK signaling is regulated by cGMP-dependent protein kinases upon activation by cCMP. Hence, cCMP could potentially act as a second messenger in the cAK/cGK and MAPK signaling pathways and play an important role in physiological processes of the jejunum.
